# Sorption of Molybdates and Tungstates on Functionalized Montmorillonites: Structural and Textural Features

**DOI:** 10.3390/ma12142253

**Published:** 2019-07-13

**Authors:** Magdalena Tuchowska, Barbara Muir, Mariola Kowalik, Robert P. Socha, Tomasz Bajda

**Affiliations:** 1 Faculty of Geology, Geophysics and Environmental Protection, AGH University of Science and Technology, al. Mickiewicza 30, 30-059 Krakow, Poland; 2 Jerzy Haber Institute of Catalysis and Surface Chemistry, Polish Academy of Sciences, Niezapominajek 8, 30-239 Krakow, Poland

**Keywords:** smectite, heavy metals, surfactants, sorbent

## Abstract

Montmorillonite—the most popular mineral of the smectite group—has been recognized as a low-cost, easily available mineral sorbent of heavy metals and other organic and inorganic compounds that pollute water. The aim of this work was to determine the sorption mechanism and to identify the reaction products formed on the surface of montmorillonite and organo-montmorillonite after sorption of molybdates (Mo(VI)) and tungstates (W(VI)). Montmorillonites are often modified to generate a negative charge on the surface. The main objective of the study was to investigate and compare the features of Na-montmorillonite (Na-M), montmorillonite modified with dodecyl trimethyl ammonium bromide (DDTMA-M), and montmorillonite modified with didodecyl dimethyl ammonium bromide (DDDDMA-M) before and after sorption experiments. The material obtained after sorption was studied by X-ray diffraction (XRD), Fourier-transform infrared spectroscopy (FTIR), scanning electron microscopy (SEM), and X-ray photoelectron spectroscopy (XPS). The XRD pattern showed the presence of a new crystallic phase in the sample that was observed under an SEM as an accumulation of crystals. The FTIR spectra showed bands related to Mo–O and W–O vibration (840 and 940 cm^−1^, respectively). The obtained results suggest that molybdenum(VI) and tungsten(VI) ions sorb onto the organo-montmorillonite in the form of alkylammonium molybdates and tungstates.

## 1. Introduction

Molybdenum (Mo) and tungsten (W) are proven to be important environmental contaminants. The understanding of the sorption mechanism of molybdenum and tungsten on mineral sorbents can enable the development of methods for their effective removal from aqueous solutions and their subsequent management. Mo and W play an important role in the chemical industry with the following applications:

- Molybdenum is used as an alloying agent; as electrodes for electrically heated glass furnaces and fire hearths; in nuclear energy applications; as a catalyst in the refining of petroleum; in radio and light bulbs as a coupling element; and as flame- and corrosion-resistant coatings for other metals.
- Tungsten is used as cemented carbide; as alloys; in electronics and electrical industries; in chemical applications; and as glass-to-metal seals. Tungsten oxides have two unique properties: intercalation and polycondensation. Thus, there is much opportunity for tungsten to find application in a fuel cell or energy-saving technologies in the future.

Mo and W are widely distributed in nature and occur in the form of various minerals, with the most common being molybdenite (MoS_2_), wulfenite (PbMoO_4_), powellite (CaMoO_4_), wolframite ((Fe,Mn)WO_4_), pinalite (Pb_3_WO_5_Cl_2_), and ferberite (FeWO_4_). Weathering of rocks and sediments rich in Mo and W has resulted in their high concentration in groundwater [1]. Moreover, the concentrations of Mo and W in the environment are significantly enhanced by anthropogenic inputs from coal-resource development, fly ash, sewage sludge, and hard rock mining activity [2]. The environmental behavior of aqueous solutions containing molybdenum and tungsten is very complex because Mo(VI) and W(VI) anions occur as a monomer only in alkaline or neutral solutions. Under even slightly acidic conditions, they tend to polymerize in the form of isopoly molybdates and tungstates with possible biotic toxicity implications [3,4]. Molybdates and tungstates usually coexist in the contaminated water, therefore, it is essential to understand Mo(VI) and W(VI) interactions in wastewater and to find an approach to efficiently remove these contaminations.

Smectites are a diverse group of clay minerals with a 2:1 layer silicate structure. Montmorillonite is the most common member of the smectite group, which has excellent sorption capacity for many heavy metals [5,6,7]. A distinctive feature of the smectite group that determines its sorption potential is that water and other polar molecules can—by entering between the unit layers—cause the structure to expand in the direction normal to the basal plane. The smectite group is characterized by an overall negative charge. Therefore, all cations are easily absorbed onto their surface [8]. These properties are determined by substitutions located in the tetrahedral and octahedral smectite layers, as well as by incomplete negative charges located on the edges of crystallites—adjacent to oxygen atoms and OH^−^ groups. However, natural smectites exhibit a weak affinity for the anionic forms of metals, which limits their use [9]. Modification of smectite with organic compounds such as quaternary ammonium salts yields a material with improved sorption properties in terms of anionic form [6,10,11,12].

To date, only the adsorption of molybdates on goethite and gibbsite has been extensively studied [13]. However, in recent years, new, functionalized materials such as magnetic macroporous cross-linked copolymers of glycidyl methacrylate [14] or thiol-containing organic molecule of pyrite [15] have become increasingly popular in the immobilization of molybdates from aqueous solutions. In addition, an evaluation of the adsorption of mono- and polytungstates onto selected soil minerals (gibbsite, birnessite, kaolinite, illite, and montmorillonite) has been described by Sen Tuna [4] and Iwai and Hashimoto [7]. Tungstate has also been shown to adsorb strongly onto iron oxyhydroxide mineral surfaces [16,17,18,19]. Other studies on the sorption of molybdenum and tungsten on a variety of materials have also been reported [20,21,22,23,24]. However, despite many studies on the immobilization of molybdenum and tungsten on different sorbents, there is still a lack of information about the sorption of mono- and polymolybdates and tungstates onto modified clay, particularly in terms of sorption mechanisms.

The aim of this paper is to investigate the structural and textural features of organically-modified montmorillonites with adsorbed anions. In our earlier studies, we analyzed the influence of surfactant amount, kinetics, pH, and initial concentration of Mo, W on the sorption of molybdates and tungstates by organo-smectites [12]. However, the proper understanding of the changes in structural and textural properties of used sorbents are crucial to finding an effective method for the disposal of both compounds. An additional challenge is to identify conditions in which the stable and nontoxic form of Mo(VI) and W(VI) will be achieved after the sorption process. This paper presents the results obtained after studying the features of organically-modified montmorillonite with absorbed molybdate and tungstate anions.

## 2. Materials and Methods

### 2.1. Materials

In this study, bentonite from the Jelšovy Potok in Slovakia, which is rich in the montmorillonite phase, was used. The preparation of the final sample consisted of several stages. First, the clay-size fraction (<2 μm) was separated from bentonite by a sedimentation process [25]. XRD confirmed that the only phase occurring after the separation procedure was Ca-montmorillonite and that all impurities from the initial sediment had been removed [12]. A strong 001 diffraction peak at 15.2 Å indicates that Ca^2+^ is the predominant cation in the montmorillonite interlayer positions [25,26]. Finally, sodium montmorillonite was prepared by introducing sodium ions into the ion-exchange positions (the source of sodium was NaCl). The procedure was described in detail by Muir et al. [12]. The mineralogical composition examined by XRD confirmed that Na-M was obtained as the modification shifted the 001 diffraction peak to 12.4 Å [26].

### 2.2. Montmorillonite Modification

Na-M was modified with organic salts, namely dodecyl trimethyl ammonium bromide (DDTMA-Br) and didodecyl dimethyl ammonium bromide (DDDDMA-Br), in the amount of 1.0 of cation exchange capacity (CEC) based on the procedure used by Bajda and Kłapyta [6]. All chemicals used were of analytical grade and were purchased from Sigma-Aldrich Co., Poznan, Poland. The samples of organo-montmorillonite were prepared by mixing 50 g of Na-M with 1000 g of the surfactant solutions at concentrations of 1.0 of CEC. After 8 h of stirring at 70 °C, the samples were centrifuged, washed 3 times with distilled water and then one time with hot ethanol, and dried at 40 °C. A similar procedure was well documented and proved to be highly effective [25]. The efficiency of the modification was determined by comparing CHN content in samples before and after modification with organic surfactants. Surfactants used in the modification process are mainly composed of carbon, hydrogen and nitrogen. By comparing the contents of C, H, and N in the dried samples before and after the modification process, the amount of surfactant adsorbed on montmorillonite was calculated. The difference in the percentage can be easily converted to the amount of adsorbed C, H, and N [mg/100 g Na-M]. All calculations were performed using the following formula: (e.g., DDTMA):

(1) $${\mathrm{DDTMA}}_{\mathrm{ad}}=\frac{{\mathrm{CHN}}_{\mathrm{difference}}}{\mathrm{mDDTMA}}$$

DDTMA_ad_—the amount of DDTMA (mmol) adsorbed on 100 g of Na-M [mmol/100 g Na-M];
CHN_difference_—the difference in the sum of carbon + hydrogen + nitrogen before and after modification [mg/100 g Na-M]. The molar fraction of each element in the surfactant was considered during the calculation.
mDDTMA—the mMolar mass of DDTMA.

Obtained sorbents were named as follow:

▪ Na-montmorillonite: Na-M
▪ montmorillonite modified with dodecyl trimethyl ammonium bromide: DDTMA-M
▪ montmorillonite modified with didodecyl dimethyl ammonium bromide: DDDDMA-M

The efficiency of modification of montmorillonite was 1.0 CEC for DDTMA-M and 0.9 CEC for DDDDMA-M. 

### 2.3. Sorption

The sorption properties were tested under static conditions. All experiments were carried out at room temperature (20 ± 2 °C). In all experiments, 100 mg of Na-M or each organo-Na-M was placed in 8 cm^3^ test tubes and 5 cm^3^ of the solution with a concentration of 50 mM of Mo(VI) or W(VI) or 50 mM of Mo(VI) + 50 mM of W(VI) and constant pH 4 was added. The sources of molybdate and tungstate ions were Na_2_MoO_4_•2H_2_O and Na_2_WO_4_•2H_2_O, respectively. The pH level was controlled with 0.1 M HNO_3_ and 0.1 M KOH.

Each sample after sorption was named using the following formula (e.g., Na-M):

▪ Na-M sample with absorbed Mo(VI) ions: Na-M-Mo
▪ Na-M sample with absorbed W(VI) ions: Na-M-W
▪ Na-M sample with absorbed Mo(VI) and W(VI) ions: Na-M-MoW

The sorption of Mo(VI) and W(VI) was determined by comparing the initial and equilibrium concentrations after 24 h under stirring, followed by centrifugation for 10 min at 14,000 rpm. After all the sorption experiments were completed, equilibrium pH and concentrations were measured.

Previous studies proved that the maximum sorption occurred at a pH below 5 and that the sorption of Mo and W decreased with an increasing pH [12]; thus, all the experiments were conducted using a solution with pH 4. The experiments have been conducted on 15 identical samples of each sorbent (Na-M, DDTMA-M and DDDDMA-M) at the same time. The pH value was measured in every fifth of them.

### 2.4. Methods

The mineralogical composition was determined by XRD (SmartLab RIGAKU diffractometer with CuKα radiation and a graphite monochromator, Tokyo, Japan) in conjunction with standard cation saturation procedures for the identification of expandable phyllosilicates [26]. The measurements were conducted in the 3 to 30° 2Θ range with a step size of 0.05° 2Θ. For the determination of C, H, and N content in the samples before and after modification, an Elemental Vario EL III CHNS automatic analyzer (Milan, Italy) was used. The specific surface area and porosity were determined from N_2_ gas adsorption/desorption isotherms at 77 K using an ASAP 2020 apparatus (Micromeritics, Norcross, GA, USA). The samples were outgassed for 24 h at 373 K. The BET equation was used for the specific surface area calculations (S_BET_) [27]. The total pore volume was calculated from the amount of N_2_ adsorbed at a relative vapor pressure (P/P0) ~0.99. The volume of micropores was calculated by applying the Dubinin–Radushkevich method [28]. The mesopore volume was determined from the adsorption branch of the isotherms by using the BJH (Barrett–Joyner–Halenda) method [29] in the mesopore range proposed by Dubinin [28]. The macropore volume (V_mac_) was calculated using the following equation:

(2) $$V_{\mathrm{mac}}=V_{\mathrm{tot}}^{0.99}-\left(V_{\mathrm{mic}}^{\mathrm{DR}}+V_{\mathrm{mes}}^{\mathrm{BJH}}\right)$$

V_mic_^DR^—the volume of micropores
V_mes_^BJH^—the volume of mesopores

Air-dried, uncoated samples were examined by SEM using a variable pressure field emission scanning electron microscope (FEI Quanta 200, Graz, Austria) equipped with an energy dispersive spectrometer (EDS, Graz, Austria) for elemental microanalysis. The FTIR spectra were collected by a Nicolet 6700 spectrometer (Fishers, Waltham, MA, USA) using the DRIFT technique (3% wt. sample/KBr) with 64 scans at 4 cm^−1^. XPS was used for the determination of the element concentration and their oxidation states at the surface layer of the studied samples. The analysis was performed in the photoelectron spectrometer equipped with the SES R4000 (Gammadata Scienta, Uppsala, Sweden) hemispherical analyzer and dual anode source. The Mg Kα radiation (200 W) was used for the analyses. The spectra were calibrated for the maximum of C 1s core excitation at the electron binding energy (BE) of 285 eV. The analytic depth of the method was estimated at approximately 10 nm. For the quantitative analysis of Mo and W concentration in the solutions, atomic absorption spectroscopy (AAS) was used (SavantAA GBC Scientific Equipment, Braeside, Australia). The analyses were conducted using a nitrous oxide–acetylene flame. The adsorption efficiency (mmol/kg) was calculated as follows:

(3) $$\mathrm{Adsorption}=100\left(\frac{C_{0}-C_{\mathrm{eq}}}{C_{0}}\right)$$

C_0_—initial Mo(VI) or W(VI) concentration [mM]
C_eq_—the concentration of Mo(VI) or W(VI) in equilibrium solution [mM] after adsorption.

## 3. Results

### 3.1. Sorption of Mo(VI) and W(VI)

The amounts of molybdenum and tungsten ions absorbed on Na-M, DDTMA-M, and DDDDMA-M are presented in Table 1. Na-M has no sorption capacity for anions. Modification of the montmorillonite significantly improved its sorption properties. The highest efficiency in terms of the removal of Mo(VI) and W(VI) ions was found for DDTMA-M. Moreover, tungstates are removed from solutions to a much greater extent than molybdates—maximum sorption capacities were 537 and 387 mmol/kg, respectively. 

The experiments were carried out on 15 identical samples of each sorbent (Na-M, DDTMA-M and DDDDMA-M) at the same time, and the pH value was measured in every fifth of them to capture the changes in pH values (Table 2). The results clearly show that sorption processes cause the increase of pH. The reaction of molybdates and tungstates with OH^−^ groups in the structure of unmodified and modified montmorillonite resulted in alkalization of the solution.

### 3.2. XRD

**Figure 1:**
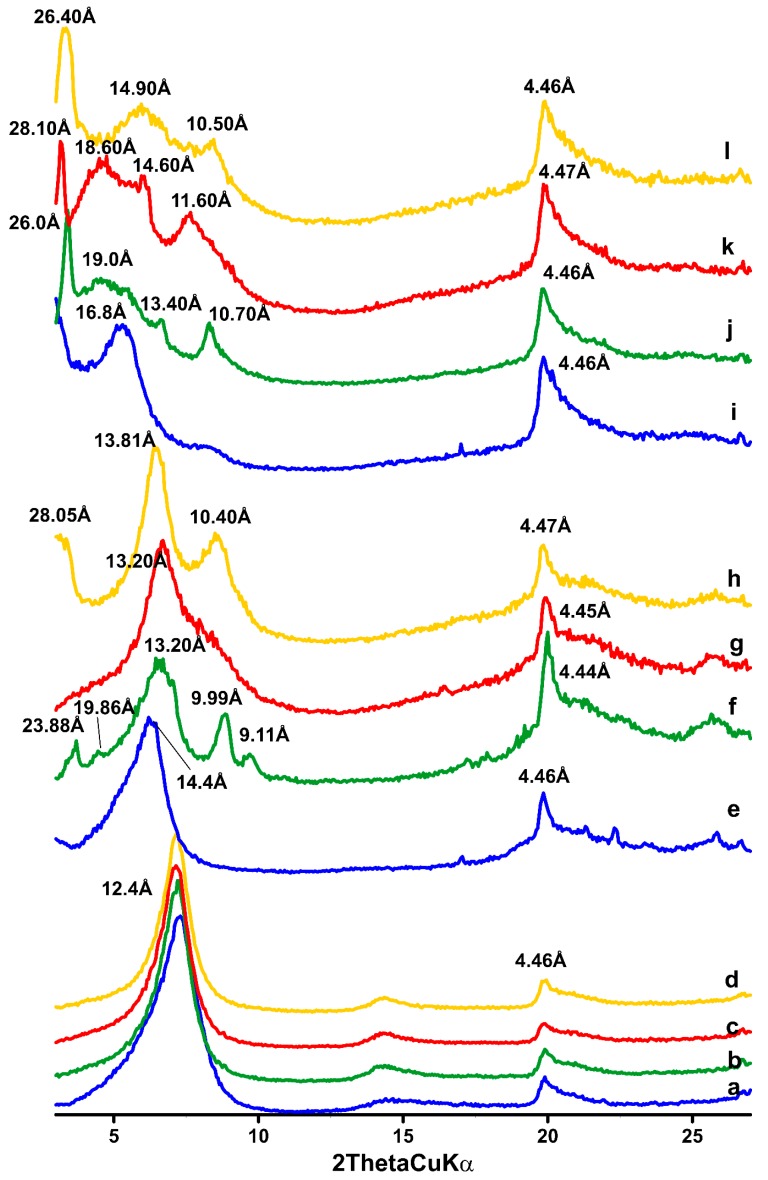
XRD patterns of samples: (**a**) Na-M, (**b**) Na-M-Mo, (**c**) Na-M-W, (**d**) Na-M-MoW, (**e**) DDTMA-M, (**f**) DDTMA-M-Mo, (**g**) DDTMA-M-W, (**h**) DDTMA-M-MoW, (**i**) DDDDMA-M, (**j**) DDDDMA-M-Mo, (**k**) DDDDMA-M-W, (**l**) DDDDMA-M-MoW.

The XRD results of montmorillonite before and after sorption experiments with organic compounds are shown in Figure 1a,e,i. The main peak (001) from montmorillonite was observed to shift toward higher interlayer distances. The peak corresponding to the interlayer space (12.4 Å) indicates the presence of sodium cations at the ion-exchange positions of montmorillonite. Reflection 4.46 Å comes from the plane family (020) of montmorillonite [30]. The intercalation of large DDTMA and DDDDMA cations resulted in an increase in the interlayer spaces of montmorillonite. The modification of montmorillonite with DDTMA increased the distance to 14.4 Å, while the addition of DDDDMA caused an increase up to 16.8 Å.

XRD of Na-M after metal sorption experiments did not show changes in the patterns (Figure 1a–d). The characteristic montmorillonite peaks of 12.4 Å and 4.46 Å that correspond to planes 001 and 020, respectively, were still present [26]. There are no new phases that could generate peaks on the diffractogram. For DDTMA-M with Mo and W adsorbed (Figure 1e–h), there were visible changes in the diffractograms. After sorption of Mo(VI), new peaks were found on the diffractograms: 23.88 Å, 19.86 Å, 9.99 Å, and 9.11 Å (Figure 1f). On the DDTMA-M-W diffractogram, no additional peaks are found (Figure 1g). Analysis of the DDTMA-M-MoW pattern confirmed the presence of additional peaks at 28.05 Å and 10.4 Å (Figure 1h). The peak corresponding to the d_001_ value in montmorillonite is visible in each diffractogram, but its value varied. On the DDTMA-M diffractogram (Figure 1e), the peak value is 14.4 Å. The sorption of Mo(VI) and W(VI) on DDTMA-M results in the peak position from shifting from 14.4 Å (DDTMA-M) to 13.2 Å (DDTMA-M-Mo), 13.2 Å (DDTMA-M-W), and 13.81 (DDTMA-M-MoW). This could be related to precipitation of Mo-DDTMA and W-DDTMA resulting in reorganization of the positions of metal ions and organic cations in the spaces of montmorillonite, leading to decrease of its d-value.

Diffractograms of DDDDMA-M with adsorbed molybdenum and/or tungsten ions are shown in Figure 1i–l. All the diffractograms contain additional peaks. For DDDDMA-M-Mo, a sharp peak is seen. This peak corresponds to a distance d equal to 26 Å, followed by a wide peak with a value of 19 Å and two successive peaks that are reflexes from the planes 13.4 Å and 10.7 Å (Figure 1j). Diffraction pattern for DDDDMA-M-W also contains four additional peaks corresponding to distances d of 28.1 Å, 18.6 Å, 14.6 Å, and 11.6 Å (Figure 1k). For DDDDMA-M-MoW, three additional peaks at 26.4 Å, 14.9 Å, 10.5 Å are visible (Figure 1l). Analysis of diffractograms of DDDDMA-M with absorbed ions (Figure 1i–l) revealed some similarity in the occurrence of four peaks at similar values of the d-value. The first sharp peak in the 2Ɵ range of 3.15 to 3.4° occurs in all the diffractograms (Figure 1i–l). The next peak for the values 4.65–4.75 of the angle 2Ɵ occurs for sorbent with absorbed Mo(VI) and W(VI). On the diffractograms of DDDDMA-M after sorption experiments, peaks in the 2Ɵ range 5.95–6.6° are visible. The last comparable range of additional peaks is visible in the 2Ɵ range of 7.65–8.45°. The diffractogram of DDDDMA-M shows a peak at 16.8 Å originating from the d(001) plane of montmorillonite [26]. In the remaining diffraction patterns, no such clear peaks are observed for similar values of the angle 2Ɵ. It can be assumed that peaks 19 Å and 18.6 Å correspond to peaks from the characteristic plane (001) of montmorillonite [26].

### 3.3. FTIR

**Figure 2:**
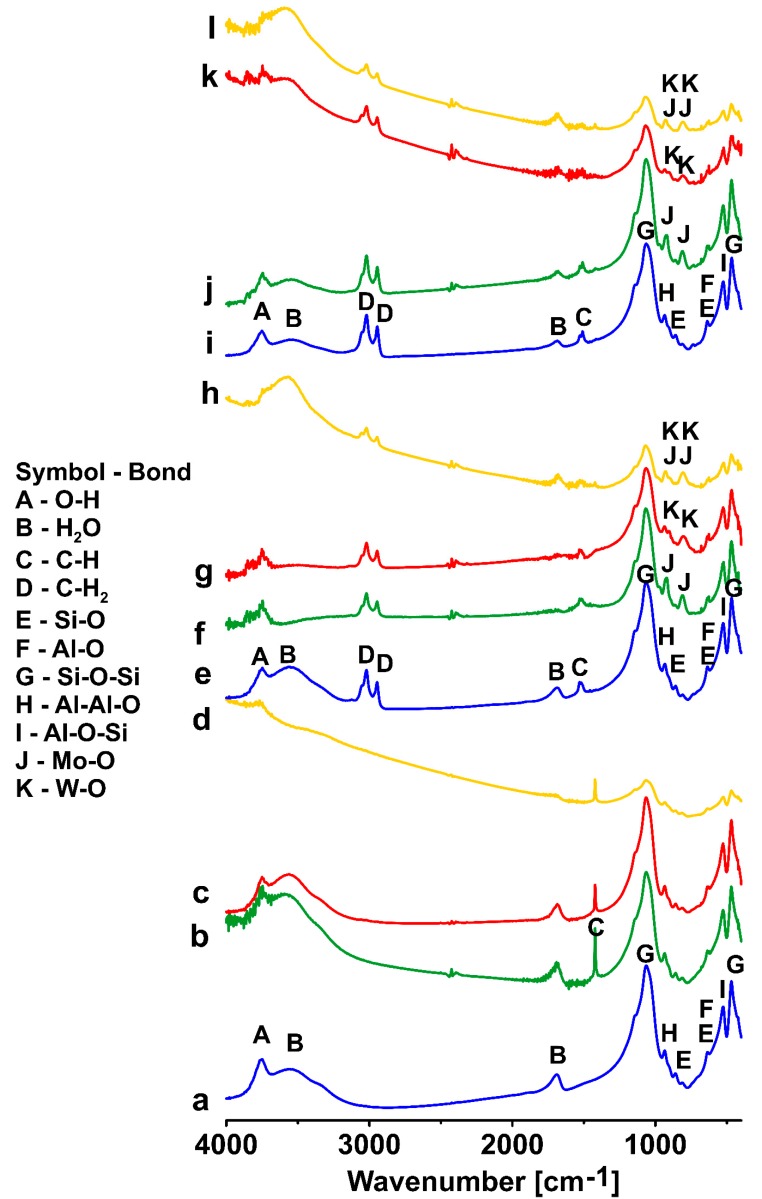
FTIR spectra of samples: (**a**) Na-M, (**b**) Na-M-Mo, (**c**) Na-M-W, (**d**) Na-M-MoW, (**e**) DDTMA-M, (**f**) DDTMA-M-Mo, (**g**) DDTMA-M-W, (**h**) DDTMA-M-MoW, (**i**) DDDDMA-M, (**j**) DDDDMA-M-Mo, (**k**) DDDDMA-M-W, (**l**) DDDDMA-M-MoW.

Major bands in MIR spectra of montmorillonite modified with DDTMA and DDDDMA, which are ascribed to bonds’ vibrations in the structure of the examined mineral, are associated with the vibration of Si-O-Si and Si-O-Al bridges and are presented in the range 1120–470 cm^−1^ (Figure 2a–l). In addition to the bands associated with OH^−^ groups (3748 cm^−1^—stretching vibrations) and water (3537 cm^−1^—tensile vibrations; 1637 cm^−1^—deformation vibrations), the tensile vibrations related to Si-O-Si bonds (1045 cm^−1^) are also visible. The bands in the 910 cm^−1^ range correspond to the deformation vibrations of the Al-Al-O bond, while the stretching vibrations of Si-O bonds cause the presence of the 819 cm^−1^ and 773 cm^−1^ bands. Some of the bands (615 cm^−1^) are derived from both Al-O and Si-O bonds. Bands corresponding to the deformation vibrations of Al-O-Si and Si-O-Si bonds are visible at approximately 500 cm^−1^ and 460 cm^−1^, respectively. The spectra of the modified material (DDTMA-M and DDDDMA-M) show two distinct bands in the 3000–2800 cm^−1^ range (Figure 2e,i), which are associated with anti-symmetric and symmetric tensile vibrations of methylene groups—CH_2_ [31]. A small band associated with bending vibrations of C-H bonds was observed at around 1470 cm^−1^ [32]. The strong band at about 3748 cm^−1^ does not show visible modification, whereas the intensity of the broad bands in the range of 3000–3700 cm^−1^ strongly depends on the kind of the surfactant. The location of a more intense band at about 3537 cm^−1^ remained unchanged and was, therefore, independent of the kind of modification. The bands in the case of Na-smectite and smectite modified both with DDTMA and DDDDMA are located in the same position. At the same time, the bands’ intensity decreases. Similar results were found in the literature [33,34].

The spectra of Na-M-Mo, Na-M-W, and Na-M-MoW (Figure 2b–d) show no changes that could be related to the presence of new bonds and functional groups containing Mo or W. In the range 3800–3100 cm^−1^, changes are visible which are associated with OH^−^ groups. The band at about 3537 cm^−1^ is strongly dependent on the surfactant and anion type. The intensity of this band rapidly decreases from some intensity in the case of DDTMA-M to background for DDTMA-M-Mo and DDTMA-M-W. While for DDTMA-M-MoW, the intensity of the band increases compared with DDTMA-M and shifts to 3568 cm^−1^. A similar observation concerns the samples modified with DDDDMA. The band at 3537 cm^−1^, which is characteristic of DDDDMA-M is slightly shifted to 3548 cm^−1^ for DDDDMA-M-Mo and to 3558 cm^−1^ for DDDDMA-M-W but does not change the intensity. For DDDDMA-M-MoW, the intensity of the band decreases compared with DDDDMA-M and its position is 3574 cm^−1^. In the spectra of DDTMA-M with adsorbed Mo(VI) and W(VI) (Figure 2f–g), two new bands in the range of 830–940 cm^−1^ are visible. The first band at approx. 838 cm^−1^ is associated with asymmetric vibrations of the Mo-O bond in the case of sorption of Mo(VI) or W-O in the case of W(VI) [35]. The range of bands originating from Mo-O and W-O bonds is identical; therefore, detailed phase identification for the material after simultaneous sorption of molybdenum and tungsten is not possible (Figure 2h). The DDTMA-M spectra show a band of approximately 2350 cm^−1^, which probably corresponds to CO_2_, which was absorbed from the air [35]. According to the literature data, the presence of bands in the range of 840 cm^−1^ and 940 cm^−1^ indicates the precipitation of molybdenum in the form of the Mo_7_O_24_^6−^ or HMoO_24_^5−^ ion and tungsten in the form of W_7_O_24_^6−^ and H_2_W_12_O_42_^5−^ [35]. In addition to the bands associated with the new bonds, montmorillonite-derived bands are also present: OH, H_2_O, C-H_2_, CH, Si-O-Si, Al-Al-O, Si-O, Si-O-Al, and Al-O-Si. At approximately 2350 cm^−1^, a band associated with adsorbed CO_2_ is observed [35]. 

The FTIR spectra of DDDDMA-M with adsorbed Mo(VI) and W(VI) (Figure 2j–l) are similar to the analogous spectra obtained for DDTMA-M. In the spectra, the presence of the same bands originating from molybdenum and tungsten can be observed, regardless of the type of surfactant used to modify the montmorillonite.

### 3.4. BET

Low-temperature nitrogen adsorption (N_2_-BET) tests provided information on the textural parameters of sorbents. The specific surface areas of BET for Na-M, DDTMA-M, and DDDDMA-M is 70.7, 3.7, and 3.1 m^2^/g, respectively. The total pore volumes of Na-M, DDTMA-M, and DDDDMA-M are 0.071, 0.028, and 0.023 cm^3^/g, respectively. In Na-M, a slightly higher proportion of mesopores (51%) than micropores (46%) was noted. DDTMA-M is mainly mesoporous (57%); however, a significant predominance of macropore (39%) over micropores (4%) was also observed. For DDDDMA-M, these values are similar (mesopores: 52%, macropores: 44%, micropores: 4%). 

**Figure 3:**
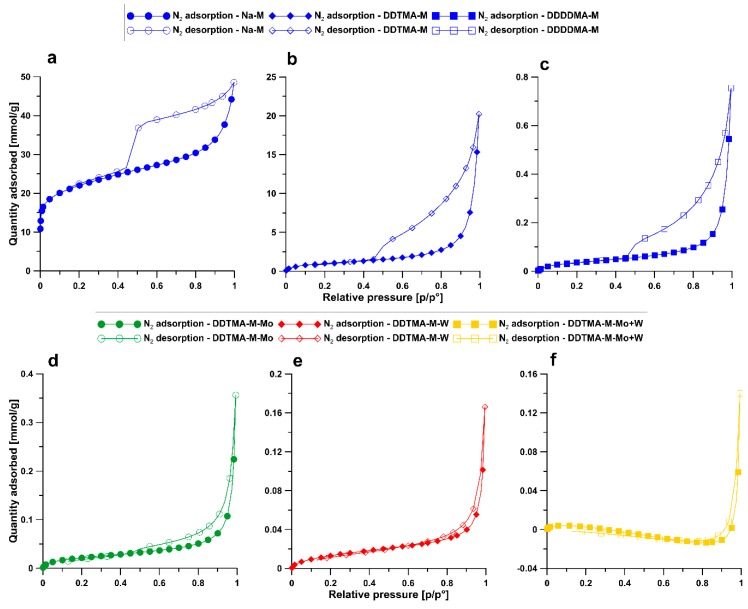
Nitrogen adsorption/desorption graph of samples: (**a**) Na-M, (**b**) DDTMA-M, (**c**) DDDDMA-M, (**d**) DDTMA-M-Mo, (**e**) DDTMA-M-W, (**f**) DDTMA-M-MoW.

On the basis of the low-temperature adsorption and desorption of nitrogen, sorption/desorption isotherms were constructed (Figure 3a–f). The IUPAC classification distinguishes I–VI types of adsorption isotherms. The isotherm shape of all sorbents corresponds to a type II isotherm [36]. For DDTMA-M (Figure 3b) and DDDDMA-M (Figure 3c), the inflection point is not clear. The hysteresis shape for Na-M, DDTMA-M, and DDDDMA-M is determined as type H3.

A nitrogen sorption/desorption isotherm was constructed for DDTMA-M with absorbed ions. The shape of the isotherms of all sorbents presented in Figure 3d–f corresponds to type II isotherms [36]. Hysteresis loops are visible on all plots. Their shape is defined as type H3.

Textural tests were carried out only for DDTMA-M with absorbed Mo(VI) and W(VI), as it was proven to be the most effective sorbent for the analyzed ions. The results are presented in Table 3. The BET specific surface area for materials with absorbed molybdenum and tungsten ions is low. It can be observed that the simultaneous adsorption of both ions had the greatest influence on the specific surface area. The resulting phase occupied more space, thus preventing the probe from penetrating the pores in the montmorillonite. The sorption of Mo(VI) and W(VI) on DDTMA-M also affected the total pore volume and the proportion of individual pore classes. DDTMA-M before the sorption experiment was defined as a mesoporous material because of the significant proportion of mesopores in the total pore volume. After the experiment of sorption of W(VI), as well as both Mo(VI) and W(VI), DDTMA-M became a macroporous material. Only the material with absorbed Mo(VI) was characterized by the same proportion of both meso- and macropores.

### 3.5. XPS

The XPS analysis enabled the determination of the chemical composition and bonds present in the sample based on the energy of the bonds. The surface concentrations of the elements in the analyzed samples are shown in Table 4. The Na-M sample surface was mainly composed of the aluminum and silicon oxide phase. Additionally, sodium, calcium, magnesium, and a small amount of nitrogen were present. Carbon contamination of the Na-M surface was low considering the powder shape. The presence of magnesium may have been caused by substitutions in the structure of montmorillonite—magnesium often substitutes aluminum in octahedral layers or may occur as a non-replaced cation at ion-exchange positions [37]. Carbon and nitrogen could be absorbed from the air.

**Figure 4:**
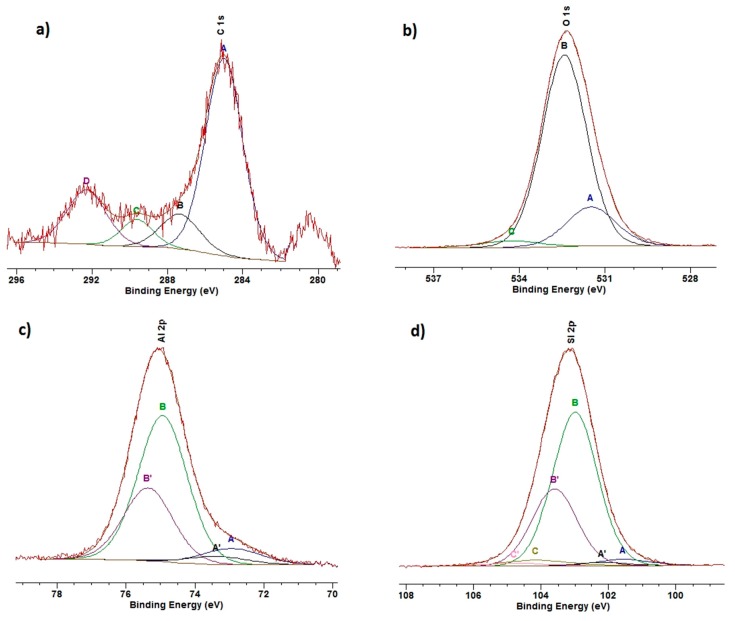
High-resolution XP spectra of Na-M: (**a**) C 1s, (**b**) O 1s, (**c**) Al 2p, (**d**) Si 2p.

The high-resolution C 1s spectrum (Figure 4a) revealed adsorbed organic compounds (A, B, and C components) and carbonates (D component (18.2%) at 292.3 eV). The A spectrum component (62.9%) at BE of 285.0 eV was assigned to aliphatic chains [38]. The broad B component (11.5%) at 287.3 eV was ascribed to the sum of alcohol and carboxyl species, and the C peak (7.4%) at 289.6 eV to carboxyl or peptide groups. The analysis of O 1s core excitation showed that the studied surface contained silica-type groups (BE of 532.4 eV) as the majority (78.8%) of surface compounds (Figure 4b). The A spectrum component (17.8%) at 531.5 eV can be assigned to hydroxyl groups and/or the oxygen bond similar to that in alumina. The C peak (3.4%) at BE of 534.3 eV confirmed that the surface contains a small amount of adsorbed organic species. The deconvolution of the Al 2p spectrum into two doublets revealed two aluminum states (Figure 4c). The main (88.3%) Al 2p_3/2_ peak (B) at 72.9 eV was assigned to the Al-O bonding in the layered mineral structure. The lower (11.7%) spectrum component at 72.9 eV was also ascribed to Al-O bonding, but in the surrounding of lower electronegativity such as that in Al_2_O_4_^2^. The Si 2p line was deconvoluted into three doublets (Figure 4d). The main spectrum component (90.5%) at 103.0 eV confirmed that the Na-M surface was composed of silica-like species. Moreover, 4.4% of overall Si 2p intensity (BE of 101.6 eV) was assigned to the bonding similar to those in silicates. The C peak (5.1%) at 104.2 eV revealed Si-OH bonding, thus confirming some surface hydroxylation [38,39].

In the case of the samples treated with DDTMA and Mo(VI) and/or W(VI) ions, the XPS analyses showed a pronounced increase in carbon concentration at the treated surfaces when compared to Na-M (Table 4). The amount of carbon was larger in the case of Mo(VI)-containing systems than in W(VI) ones. In the case of Mo(VI) ions, better coverage of the surface by DDTMA-M was supported by lower oxygen, silicon, aluminum, and sodium concentrations. The amount of adsorbed Mo(VI) ions was relatively large (7.8 at%). In the case of W(VI) ion adsorption, the resulting concentration (3.3 at%) was lower than twice that of the Mo(VI) system. The mixed Mo(VI) and W(VI) system showed similar amounts (app. 3 at%) of both ions at the treated surface. It is worth mentioning that at all treated surfaces, the calcium concentrations were found to be below the level of detection.

**Figure 5:**
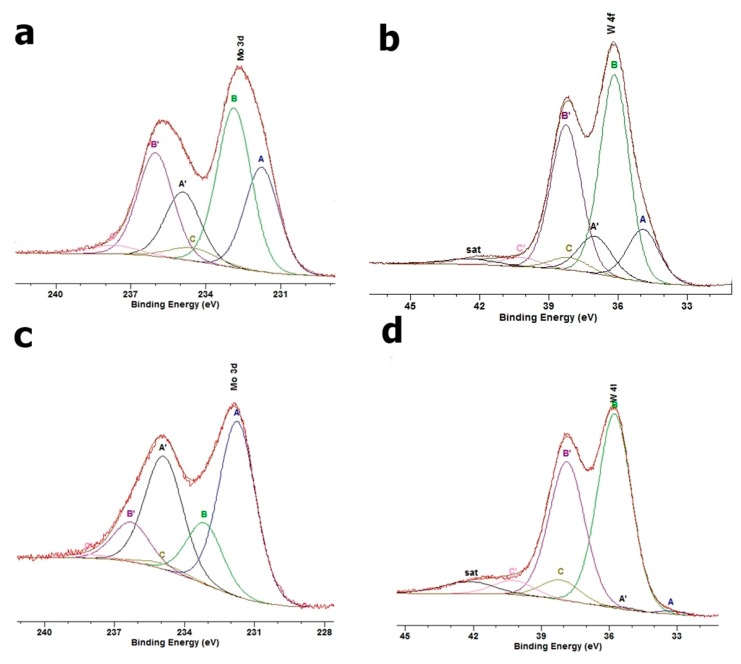
High-resolution XP spectra of (**a**) Mo 3d in a DDTMA-M-Mo, (**b**) W 4f in a DDTMA-M-W, (**c**) Mo 3d in a DDTMA-M-MoW, (**d**) W 4f in a DDTMA-M-MoW.

The electronic states of adsorbed elements were also analyzed by XPS. In the case of the DDTMA-M-Mo system (Figure 5a), the Mo 3d spectrum was deconvoluted into three doublet states. The BE of Mo 3d_5/2_ excitation for the A component (37.1%) at 231.8 eV indicated the presence of Mo^5+^ ions in the form of oxide. The most intensive component (56.6%, 232.9 eV) is, however, assigned to Mo(VI) bonded to oxide ions. Additionally, a small amount (6.3%, 234.5 eV) of Mo^6+^-OH type of bonding was found, which indicated some surface hydroxylation [38]. In the DDTMA-W system, the W 4f spectrum was deconvoluted into three doublets (Figure 5b). The most intensive W 4f_7/2_ peak (73.4%) at 36.2 eV was assigned to W(VI) in the oxide form. The A peak (20.8%) at BE of 34.9 eV was ascribed to W^4+^ ions in an oxide surrounding and the C spectrum component (5.7%) at 38.1 eV indicated W(VI) bonded to hydroxyl groups [38]. In the DDTMA-M-MoW system, the Mo 3d spectrum changed its envelope as compared to the DDTMA-M-Mo system. A large increase (from 37.1% to 72.4%) of A component at 231.7 eV indicated a reduction in the majority of molybdenum ions to Mo(IV) in the oxide form. The remaining Mo(VI) oxide form contributed to 24.8% of the overall Mo 3d spectrum intensity. The hydroxylated part of the Mo(VI) remained similar (2.8%) to that in the DDTMA-M-Mo system. The W 4f excitation showed that in the mixed system, the majority (88.8%) of tungsten ions were present as W(VI) in the oxide surrounding. The amount of W(IV) was low (1.0%), and the amount of hydroxylated W(VI) species was almost twice (10.2%) that in the DDTMA-M-W system. The latter found confirmation in respective O 1s spectrum where surface hydroxylation was larger than at Na-M sample surface. In the case of DDTMA-M-Mo and DDTMA-M-W systems, the reduction in the number of Mo(VI) or W(VI) ions observed at the DDTMA-M surfaces can be a result of either bonding to nucleophilic centers (resulting in electronic shift similar to reduction) or real ion reduction due to surface alkalinity and carbonaceous species (C-OH, C=O) present at the surface.

### 3.6. SEM

**Figure 6:**
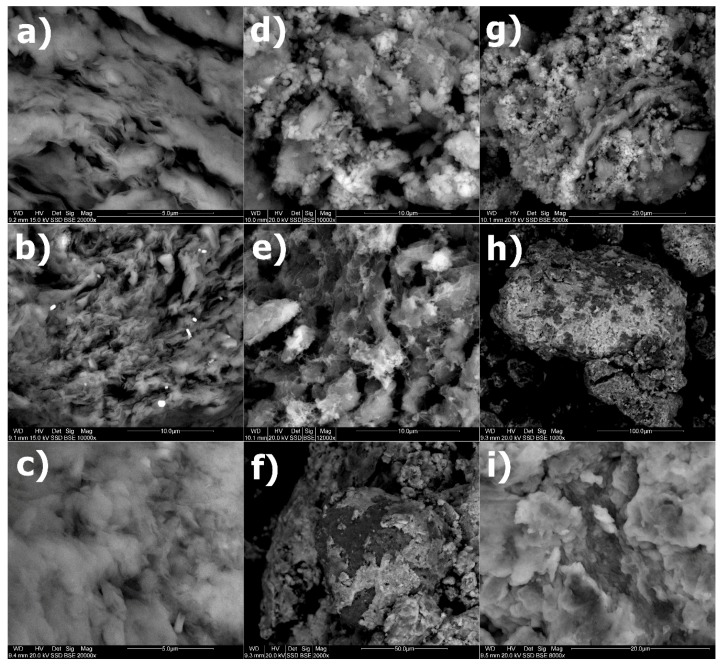
SEM images of samples: (**a**) Na-M, (**b**) DDTMA-M, (**c**) DDDDMA-M, (**d**) DDTMA-M-Mo, (**e**) DDTMA-M-W, (**f**) DDTMA-M-MoW, (**g**) DDDDMA-M-Mo, (**h**) DDDDMA-M-W, (**i**) DDDDMA-M-MoW.

The SEM image for Na-M (Figure 6a) shows the surface morphology of the sorbent. A layer-like texture resembling a tissue layer is visible. The separation zones are distinct. The morphology of the samples after the modification did not change; thus, the typical morphology of montmorillonite is evident. Single precipitations of DDTMA-Br and DDDDMA-Br surfactant are visible in images for DDTMA-M (Figure 6b) and DDDDMA-M (Figure 6c), respectively. The SEM method enabled observation of the morphology of the sample with absorbed Mo(VI) and W(VI). The SEM images of DDTMA-M-Mo (Figure 6d) and DDTMA-M-W show new phases, that appear on the montmorillonite surface. A similar image was obtained for the montmorillonite modified with DDDDMA-Br surfactant. In the SEM image for DDDDMA-M-Mo (Figure 6g), larger forms of precipitates were visible than those present on DDTMA-M-Mo. In addition, for DDDDMA-M-W (Figure 6h), the precipitates on the organo-mineral surface are visible. The SEM image for a DDDDMA-M sample with absorbed Mo(VI) and W(VI) (Figure 6i) shows crystallized forms, however, despite the crystallization, the montmorillonite tissue structure remained intact.

## 4. Discussion

The sorption experiments revealed that Na-M has no sorption capacity for anions due to the negative charge of the surface; this finding is consistent with the observation of Murray [40]. However, surface modification with surfactants caused a change in the surface charge and an increase in the sorption capacity of Na-M relative to anions. The negative surface of Na-M can be easily transformed into a positively charged one by replacing the metal ions with large organic cations, such as DDTMA and DDDDMA. The modification process includes the introduction of organic cations into ion-exchangeable positions. Thus, the negative electrostatic charge of structural layers are naturally compensated by cations adsorbed in the interlayer space [41]. DDTMA-M is proven to have a better sorption capacity than DDTMA-M. It can be explained by the differences in the structure of these molecules. A previous study indicated that with an increasing amount of carbon chain and its length, the sorption efficiency decreases [42], which is also proven by the results obtained in this study.

The sorption of Mo(VI) and W(VI) resulted in an increase in pH values. The reaction of molybdates and tungstates with OH^−^ groups in the structure of unmodified and modified montmorillonite resulted in alkalinization of the solution. Some researchers [43,44] indicate that low-molecular-weight substances containing diol-groupings can form complexes with metal-oxyanions. For Mo and W, these hydroxy compounds can form bi- or poly-nuclear complexes. OH^−^ groups might form polyol-complexes with Mo and W anions [44]. However, it is also possible that molybdates and tungstates may be present in the solution in the form of polyions developed from metal, water, and OH^−^ groups [45]. During sorption, a simple form of monoion is sorbed, and OH^−^ groups remain in the solution, thus alkalizing it. The immobilization of both Mo(VI) and W(VI) in the mixed solution is difficult due to the interference of these elements. Mo(VI) and W(VI) anions occur as a monomer only in alkaline or neutral solutions. Under acidic conditions, they polymerize to the form of isopoly molybdates and tungstates causing limited sorption of these anions from mixed solutions [46,47]. The sorption of Mo(VI) and W(VI) is limited at pH > 5 and pH > 6, respectively [12]. Mo-oxyanions are less stable than W-oxyanions and are more sensitive to pH change; further, with the increasing pH (from 3.5 to 5.5), the sorption capacity of molybdates decreased more rapidly than that of tungstate [44]. This finding can explain the higher sorption capacity of W(VI) ions.

The present findings demonstrate that montmorillonites modified with DDTMA and DDDDMA are effective sorbents of Mo(VI) and W(VI). The most effective sorbent for the removal of Mo(VI) and W(VI) was DDTMA-M, which adsorbed 388 mmol/kg Mo(VI) and 537 mmol/kg W(VI). Literature data reveal, that removal of tungstate using popular sorbents such as gibbsite, goethite or birnessite was significantly lower than the results obtained in this study (201, 174 and 24 mmol/kg, respectively) [4,7]. Previous studies also show, that for some sorbents, such as ferrihydrite, the sorption capacities obtained by different researchers differ significantly, form 120 mmol/kg to 420 mmol/kg [7,48]. It was observed that popular sorbents are also less effective than organically modified montmorillonite. Goethite was able to immobilize 160 mg/kg of Mo(VI); gibbsite—200 mmol/kg; aluminum oxide—70 mmol/kg; kaolinite—1.5–3 mmol/kg; illite—3.75 mmol/kg [13,16]. Resembling sorption capacities can be observed for drinking water treatment residues—324 mmol/kg; or iron oxide-coated sand—305 mmol/kg [49,50].

The possibility of regeneration and reuse of different organically modified clay minerals have already been discussed in the literature. Desorption isotherms show apparent variability in desorption behavior among the different anions and also, among different concentrations of the same anion. In general, desorption did not appear to be completely reversible for any of the organically modified clay minerals; however, in many cases it was possible to desorb up to 70% of absorbed anion [51,52,53].

The impact of surfactants on the structure of montmorillonite is confirmed by XRD results. The modification of montmorillonite with DDTMA increased the distance to 14.4 Å, while the addition of DDDDMA caused an increase up to 16.8 Å. This difference is probably caused by the size of the surfactant molecule—DDTMA has a shorter carbon chain, while DDDDMA has two long carbon chains attached to pentavalent nitrogen. According to the literature data, the thickness of the montmorillonite unit is 9.70 Å and the molecular dimension of DDTMA is approximately 3.8 Å in height and 18.0 Å in length when the alkyl chain of the DDTMA is parallel to the plane of montmorillonite [54,55]. Thus, it is assumed that the obtained d-value indicates a horizontal arrangement of surfactant molecules in the interlayer spaces [56].

**Figure 7:**
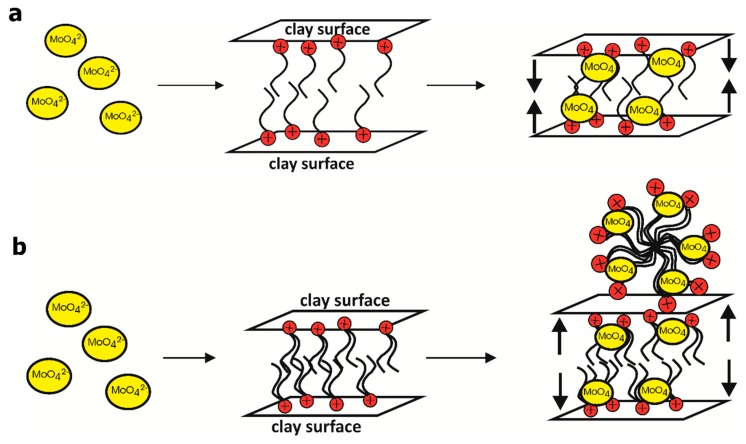
Mechanisms of immobilization of Mo(VI) onto: (**a**) DDTMA-M, (**b**) DDDDMA-M.

Poor sorption capacity of Na-M regarding anions are confirmed—there are no new phases that could generate peaks on the diffractogram. For DDTMA-M with Mo and W adsorbed, there are visible changes in the diffractograms; some additional peaks are seen, which may suggest the presence of new crystalline phases in the sample. Analysis of the DDTMA-M-MoW pattern also indicates the presence of additional peaks, which may suggest the emergence of new crystalline phases. The speculation is based on the occurrence of two distinct peaks at 28.05 Å and 10.4 Å. The peak corresponding to the d_001_ value in the montmorillonite is 14.4 Å. The shift to higher d value suggests that the sorption processes resulted in a slight reduction in the interlayer distance. We assume that the reduction of the montmorillonite interlayer distance may be caused by the reaction of Mo(VI) and W(VI) with the organic cations. The emerging phase may generate a specific arrangement in the space between the packets, which occupies less space than the organic cation or the precipitation of the phase on the surface of the organo-montmorillonite, which was also suggested by Bajda and Kłapyta [6]. Diffractograms of DDDDMA-M in which Mo and W was adsorbed contain additional peaks, which are comparable for all the diffractograms of DDDDMA-M with absorbed ions. This observation suggests the occurrence of similar phases in the structure of DDDDMA-M after sorption processes. Peaks corresponding to the characteristic plane (001) of montmorillonite (19 Å and 18.6 Å) indicate an increase in the distance between the organo-montmorillonite packets. It is probably caused by the precipitation of the phase in the interlayer space [6,57]. However, it must be taken into account, that precipitation of anion salts on the surface is also possible. Previous studies concerning the immobilization of chromates, vanadates, molybdates and tungstates onto HDTMA-modified clay minerals suggest that precipitation is probable [58,59]. Thus, we believe that similar phenomena may occur during the immobilization of molybdates and tungstates onto DDTMA-M and DDDDMA-M. However, there is no unambiguous proof of this. Mechanisms of immobilization of Mo(VI) and W(VI) are similar. The proposed mechanisms for molybdates are presented in Figure 7.

The FTIR spectra of Na-M-Mo, Na-M-W, and Na-M-MoW prove that sorption of anions did not occur. There are no changes that could be related to the presence of new bonds and functional groups containing Mo or W. However, the occurrence of new bands on the spectra of DDTMA-M and DDDDMA-M with adsorbed Mo(VI) and W(VI) allowed for the conclusion that polymerized forms of molybdenum and tungsten ions were present in the sample in the form of the Mo_7_O_24_^6−^ or HMoO_24_^5−^ ion or W_7_O_24_^6−^ and H_2_W_12_O_42_^5−^ [35]. A decrease in the intensity of the band connected with OH^−^ groups’ vibrations (the bands’ intensity for DDTMA-M and DDDDMA-M decreases compared with Na-M) indicates some reduction of interlayer water content caused mainly by surface hydrophobisation and by displacement of water molecules in the interlayer space by DDTMA+ and DDDDMA+ cations. A more visible effect in the total reduction of interlayer water content is observed for DDTMA-M and DDTMA-W. It is possible to assume that Mo(VI) and W(VI) precipitated with DDTMA in interlayer spaces of montmorillonite and completely removed the OH^−^ ions.

A significant difference in specific surface values of the modified montmorillonite is caused by blocking the access of the N_2_ probe to the pores in DDTMA-M and DDDDMA-M by large organic cations. The shape of all low-temperature sorption/desorption isotherms for all sorbents corresponds to type II [36] which suggest that sorption is multilayered and unlimited in the range of high relative pressures. The initial shape of the isotherm for Na-M indicates monolayer adsorption, and at the inflection site of the isotherm, multilayer adsorption began. The inflection points of the isotherms for DDTMA-M and DDDDMA-M are not clear. This indicates that in the beginning, the monolayers and the formation of multilayers on the surface of the adsorbent occurred [60]. In the range of relative pressures below p/p0 ~0.45, micropores are filled, and above this value, meso- and macropores are filled. For all sorbents, hysteresis loops are visible, which are directly related to the capillary action of liquid nitrogen occurring in the mesopores [36]. Type H3 of the hysteresis shape indicate that Na-M, DDTMA-M, and DDDDMA-M are materials characterized by slit-shaped pores [61,62].

A nitrogen sorption/desorption isotherm constructed for DDTMA-M also corresponds to type II isotherms [36]. In the range of low relative pressures, multilayer adsorption on the materials was found because of the lack of pronounced inflection of the sorption curves. Monolayers consisting of N_2_ molecules formed a multilayer [60]. An interesting phenomenon was observed for DDTMA-M-MoW. The sorption curve has reached values below zero. This may indicate the evolution of nitrogen from the sample during analysis. This was possible because nitrogen is one of the components of the DDTMA molecule.

Analysis of high-resolution XP spectra was performed in correlation with the Na-M surface state. The C 1s spectrum changes are the effect of surface treatment of all the analyzed samples. Two spectrum features were then observed, namely the component assigned to carbonates disappeared and an additional component appeared at approximately 283.3 eV. This additional component was ascribed to carbon–metal bonding (possibly through oxygen or nitrogen) that indicated the presence of the DDTMA-metal system at the Na-M surface. Additionally, the B peak intensity increased and the peak maximum shifted to approximately 286.5 eV, which indicated the presence of C-O and C-N bonding after the treatment. 

An analysis of O 1s core excitation revealed that the performed treatment resulted in a pronounced increase in the A peak assigned to hydroxyl groups at the cost of a decrease in the B peak (O-Si bonds). Such behavior could indicate preferential adsorption of DDTMA compounds on O-Si-bonds. The adsorption process resulted in the appearance of an additional peak at approximately 529.0 eV. This peak could indicate the presence of defected metal-oxide systems at the treated surfaces. The change in intensities of O 1s spectrum components was correlated with the metal ions used in the treatment. It was found that Mo(VI) ion adsorption resulted in a larger concentration of hydroxyl groups (lower O-Si amount) at the surface than in the case of the W(VI) ion system. This behavior indicates a different mechanism of Mo(VI) and W(VI) ion adsorption at the Na-M surface. The electronic composition of Al 2p core excitation was found to be stable and comparable to the one at the Na-M surface for all the treated surfaces. In the case of Si 2p spectra, the change caused by Mo(VI) ions was strong and resulted in an increase (from 4.4% to 22.8%) in the intensity of the component at 102.1 eV. This component was ascribed to Si-O–metal bonding and indicated adsorption of Mo(VI) ions on Si-O groups. Application of W(VI) ions in the treatment resulted in increased hydroxylation of silicate ions. When the system of both ions was used for the treatment, the Si 2p line showed mainly (95.1%) the basic silica state at the treated surface. 

The SEM method enabled the observation of new phases on the surface of modified montmorillonites with absorbed ions. In the SEM image of DDTMA-M-Mo, incrustations on the lamellar edges of montmorillonite are visible. To summarize the results so far, it is possible to state that it is an inorganic–organic salt. The source of organic salt components is DDTMA cation and inorganic molybdate ion [6,57]. In the SEM image, there are visible inlays of these salts on the surface of DDTMA-M. Their presence affected the morphology of the montmorillonite surface, and the disappearance of the tissue structure can be observed. A similar image was obtained for a DDTMA-M-W, however, the inorganic–organic salt consisting of the tungstate anion and the DDTMA cation takes the form of a “coating” on the surface of the montmorillonite. A different morphology of DDTMA-M-Mo and DDTMA-M-W is caused by the effect of different amounts of these ions being absorbed on DDTMA-M. DDTMA-M shows a higher sorption capacity for W(VI) than for Mo(VI). A large amount of adsorbed W(VI) ions resulted in the formation of inorganic–organic salt covers on the montmorillonite surface. For DDTMA-M-MoW, a similar morphology in the form of covers on the surface of DDTMA-M can be observed. The form of salt precipitations on organo-montmorillonite depends on the availability of sites for crystallization. The salt crystallized in areas where organic cations are found. On the less accessible surface, ions formed single concentrations [6,63].

In the SEM image for DDDDMA-M-Mo, larger forms of salt precipitations were visible than those present on DDTMA-M-Mo. The form of the precipitations indicates a lower availability of the montmorillonite surface to crystallize the salt; this was the case for DDTMA-M. The lower effectiveness of DDDDMA to modification of Na-M is related to the smaller amount of organic cations present in/on the organo-montmorillonite, and thus fewer available places are accessible for the crystallization of organic–inorganic salt. For DDDDMA-M-W, the precipitation of salts is visible. W(VI) sorption on DDDDMA-M was three times higher than Mo(VI) sorption. Thus, the precipitation of an inorganic–organic salt consisting of a tungstate anion occupied a larger area than the precipitation of salt with Mo(VI). The SEM image for DDDDMA-M-MoW, shows a crystallized inorganic–organic salt; however, despite the crystallization of salt, it does not has an impact the montmorillonite tissue structure.

## Figures and Tables

**Table 1:** Sorption capacities of Na-M, DDTMA-M, and DDDDMA-M regarding Mo(VI) and W(VI) ions (based on the AAS results). T = 20 °C, C_0_ = 50 mM, initial pH = 4.

Sample	Sorption Capacity [mmol/kg]			
	Mo(VI)	W(VI)	Mo(VI) + W(VI)	
			Mo(VI)	W(VI)
**Na-M**	<0.01	<0.01	<0.01	<0.01
**DDTMA-M**	387.72 ± 28.99	537.41 ± 189.36	8.01 ± 1.13	48.26 ± 4.00
**DDDDMA-M**	84.33 ± 31.47	260.67 ± 38.19	9.36 ± 0.90	54.14 ± 3.05

**Table 2:** pH values of the solutions remaining after sorption of Mo(VI), W(VI), and Mo(VI) + W(VI). Initial pH = 4, T = 20 °C, C_0_ = 50 mM.

Absorbed Ions	Sample	Sorbent		
		Na-M	DDTMA-M	DDDDMA-M
		pH Values		
**Mo(VI)**	1st	4.71	4.71	4.78
	5th	4.65	4.78	4.82
	10th	4.68	4.63	4.70
	15th	4.73	4.72	4.75
**W(VI)**	1st	5.00	5.40	5.36
	5th	4.44	5.30	5.20
	10th	5.02	5.28	5.40
	15th	5.00	5.36	5.37
**Mo(VI) + W(VI)**	1st	4.49	4.61	4.54
	5th	4.47	4.78	4.57
	10th	4.53	4.62	4.57
	15th	4.48	4.61	4.62

**Table 3:** The textural parameters of DDTMA-M-Mo, DDTMA-M-W, and DDTMA-M-MoW.

Sample	BET Surface Area [m^2^/g]	Total Pore Volume [cm^3^/g]	Volume of Micropores [cm^3^/g]	Volume of Mesopores [cm^3^/g]	Volume of Macropores [cm^3^/g]
**DDTMA-M-Mo**	1.8	0.011	0.001	0.005	0.005
**DDTMA-M-W**	1.2	0.005	<0.001	0.002	0.003
**DDTMA-M-MoW**	0.4	0.004	<0.001	0.001	0.003

**Table 4:** Atomic concentrations of elements in Na-M, DDTMA-M-Mo, DDTMA-M-W, and DDTMA-M-MoW samples.

Sample	C 1s	O 1 s	Si 2 p	Al 2p	Na 1s	N 1s	Ca 2p	Mg 2s	Mo 3d	W 4f
**Na-M**	6.3	57.0	23.8	10.2	1.1	0.2	0.2	1.3	-	-
**DDTMA-M-Mo**	52.8	28.5	6.4	1.6	0.5	1.6	-	0.7	7.8	-
**DDTMA-M-W**	32.6	40.2	15.7	5.7	0.2	1.9	-	0.4	-	3.3
**DDTMA-M-MoW**	45.4	32.9	8.4	4.1	0.2	1.6	-	1.1	3.0	3.3
